# Clinical Spectrum and Admission Correlates of Complicated Imported Malaria in Polish Travellers: An 11-Year Retrospective Analysis with Geographic Mapping of Travel Destinations

**DOI:** 10.3390/jcm15187269

**Published:** 2026-09-18

**Authors:** Damian Pikor, Jadwiga Duda, Maximilian Ciechanowski, Jacek Czepiel, Maciej Cymerys, Krzysztof Macheta, Aleksandra Wesełucha-Birczyńska, Monika Bociąga-Jasik, Małgorzata Paul

**Affiliations:** 1 Department of Internal Medicine and Metabolic Disorders, Poznan University of Medical Sciences, Przybyszewskiego 49, 60-355 Poznan, Poland; 2 Department of Tropical and Parasitic Diseases, University Clinical Hospital, Przybyszewskiego 49, 60-355 Poznan, Poland; 3 Student Scientific Association, Department of Infectious Diseases, Jagiellonian University Medical College, 30-688 Krakow, Poland; 4 Student Scientific Society, Poznan University of Medical Sciences, Rokietnicka 5, 60-806 Poznan, Poland; 5 Department of Infectious Diseases, Jagiellonian University Medical College, 30-688 Krakow, Poland; 6 University Hospital in Krakow, Jakubowskiego 2, 30-688 Krakow, Poland; 7 Faculty of Chemistry, Jagiellonian University, Gronostajowa 2, 30-387 Krakow, Poland

**Keywords:** biomarkers, chemoprophylaxis, severity-capture criteria, *Plasmodium falciparum*, sub-Saharan Africa, travel medicine

## Abstract

**Background**: Complicated malaria occurs in 15–40% of hospitalised imported cases, and European centres often define it more broadly than the WHO 2023 severe-malaria criteria, which, calibrated for semi-immune populations, may under-identify severe disease in non-immune travellers; Central and Eastern European data are limited. We examined the clinical spectrum, travel destinations, and admission correlates of complicated imported malaria in non-immune Polish travellers. **Methods**: This was a two-centre, 11-year retrospective cohort of 93 laboratory-confirmed episodes in 91 adults (December 2014–October 2025). Complicated disease was a composite of organ-specific complications on admission. We derived the exploratory nine-item IMSC-NIT severity-capture criteria, with a pre-specified analysis restricted to the four items not overlapping the outcome (age, C-reactive protein, procalcitonin, platelets). **Results**: Complicated disease affected 31/93 episodes (33.3%), exceeding the 10/93 that met the strictly assessable WHO criteria. *Plasmodium falciparum* predominated; 88.2% with documented status had used no chemoprophylaxis; age was the only independently associated variable (multiple-imputation odds ratio 1.07 per year, 95% CI 1.02–1.11). The restricted four-item score gave an area under the curve of 0.76 (95% CI 0.65–0.85; optimism-corrected 0.72). **Conclusions**: The WHO criteria alone may miss non-immune travellers with organ involvement at presentation. The IMSC-NIT is a hypothesis-generating severity-capture aid requiring external validation before clinical use.

## 1. Introduction

Malaria remains among the most consequential infectious diseases globally, with an estimated 263 million cases and 597,000 deaths in 2023 [1]. In non-endemic Europe, the European Centre for Disease Prevention and Control reported approximately 8000 EU/EEA cases annually before COVID-19, with a post-pandemic rebound from 2022 [2]. Central and Eastern European data, however, remain limited. Unlike Western European referral centres shaped by post-colonial migration, Poland has no comparable colonial legacy; its cases arise predominantly in non-immune tourists and occupational or missionary travellers, and historically numbered approximately 30–40 per year, with an upward trend paralleling increased travel to sub-Saharan Africa [3]. Such travellers lack the partial immunity of endemic populations and may present with higher parasitaemia and greater organ dysfunction than semi-immune patients [4]. This matters clinically: complicated malaria occurs in 15–40% of hospitalised imported cases depending on the definition [5,6], and European centres often operationalise it more broadly than the WHO 2023 severe-malaria criteria [7], which were calibrated for semi-immune endemic populations and may under-identify severe disease in non-immune travellers. West Africa contributes the largest share of imported *P. falciparum* [8]. Chemoprophylaxis remains the cornerstone of prevention, yet 60–90% of hospitalised imported cases in Europe have not used or completed a recommended regimen [9], and admission biomarkers such as C-reactive protein (CRP) and procalcitonin (PCT) are less well characterised against composite than single-organ endpoints [10,11]. We therefore characterised the clinical spectrum and travel destinations of imported malaria in a Polish two-centre 11-year cohort, identified admission correlates of a complicated course, and proposed a nine-item admission severity index, the Imported Malaria Severity Criteria for Non-Immune Travellers (IMSC-NIT), to complement the endemic-derived WHO criteria as a standardised severity-capture aid at admission in non-immune travellers.

## 2. Materials and Methods

### 2.1. Study Design and Setting

This was a two-centre retrospective cohort study of adult patients hospitalised for laboratory-confirmed imported malaria at the Department of Infectious Diseases, University Hospital in Kraków, and the Department of Tropical and Parasitic Diseases, University Clinical Hospital, Poznań University of Medical Sciences, Poznań, Poland. Both institutions serve as regional referral centres for tropical infections. The study period extended from December 2014 to October 2025. The study was conducted in accordance with the Declaration of Helsinki. The Bioethics Committee of Poznan University of Medical Sciences determined that the study did not require committee approval (ruling no. KB-62/26, 28 January 2026), as it comprised a retrospective analysis of routinely collected, de-identified clinical records; the Bioethics Committee for Scientific Research of Jagiellonian University Medical College issued a favourable opinion for the Krakow centre (no. 118.0043.1.48.2025, 20 February 2025). Data were processed and held locally in accordance with the General Data Protection Regulation. Analyses were specified before the inspection of outcomes; the analysis code is available on request.

### 2.2. Patients

Eligible patients were adults (≥18 years) with a discharge diagnosis of malaria confirmed by light microscopy of thick and thin blood films and a documented travel history to a malaria-endemic country. No exclusion criteria were applied beyond data availability. The analysis cohort comprised 93 malaria episodes in 91 patients; two patients each contributed two separate episodes. The selection of episodes is summarised in Figure S1.

### 2.3. Data Collection

Demographic, travel, clinical and laboratory data on admission were abstracted from medical records: *Plasmodium* species, parasitaemia, chemoprophylaxis use, Glasgow Coma Scale, full blood count, inflammatory markers (CRP, procalcitonin), liver-function tests, urea, creatinine, and documented clinical signs. Comorbidities and the reported day of symptoms were abstracted as free text where documented. Serum bilirubin, baseline (pre-admission) and serial creatinine, arterial blood gases, numeric blood pressure and dates of antimalarial initiation were not available in the source records.

### 2.4. Definitions

The primary outcome, complicated malaria, was the presence on admission of at least one of four organ-specific complications: clinician-documented renal impairment, clinical jaundice, impaired consciousness (Glasgow Coma Scale < 15) or macroscopic haemoglobinuria. Renal impairment and jaundice were recorded by the treating physicians as binary clinical assessments; because baseline and serial creatinine, urine output and serum bilirubin were not available, KDIGO staging and bilirubin-based definitions could not be applied. Identical coding rules were applied to the records of both centres during data abstraction. This operational composite was chosen by the authors to capture early multi-organ involvement in non-immune travellers and is deliberately broader than the WHO 2023 severe-malaria criteria, which use higher endemic-setting thresholds (for example, Glasgow Coma Scale < 11 and creatinine > 3 mg/dL); we used a threshold of <15 because any impairment of consciousness is clinically significant in this population. Chemoprophylaxis was classified as used, not used or unknown from documentation, and severe thrombocytopenia as a platelet count below 50 × 10^3^/µL.

### 2.5. Geographic Classification

**Figure 1:**
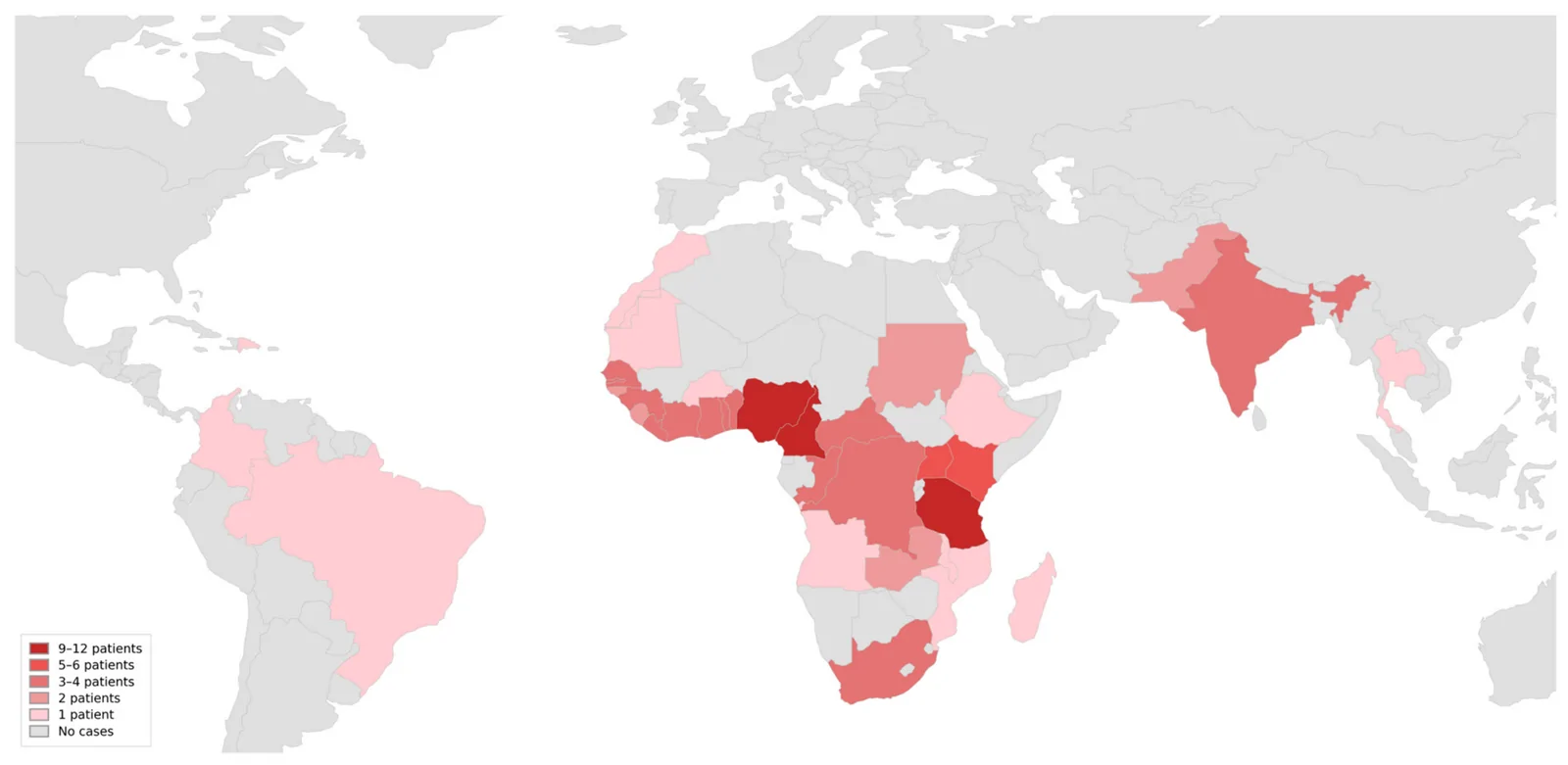
Travel destinations of imported malaria episodes (n = 93). Colour intensity reflects the number of episodes per destination country; exact counts for all 37 countries are given in Table 1.

Countries visited were extracted from the records. Table 1 and Figure 1 list every country visited, so patients with a multi-country itinerary contribute to each country and the counts sum to more than the number of patients. For the mutually exclusive regional classification, each patient was assigned to a single standard geographic region on the basis of the first listed destination; because the duration of stay and incubation could not be reconstructed for every patient, this assignment is approximate and is used only for descriptive purposes.

### 2.6. Statistical Analysis

Analyses were performed using Python 3.12 (pandas 3.0.0, scipy 1.17.0, statsmodels 0.14.6, scikit-learn 1.8.0). The exploratory derivation of the severity criteria is reported against the TRIPOD statement [12], and the observational cohort in accordance with the STROBE recommendations [13]; completed checklists are provided with the Supplementary Materials. Continuous variables are expressed as median (interquartile ranges, IQR) and categorical variables as counts (%), with denominators reflecting available data. Groups were compared with the Mann–Whitney U and Fisher exact tests, with Benjamini–Hochberg control of the false-discovery rate across the admission-variable family; complication rates across regions were compared with the chi-squared test. C-reactive protein and procalcitonin were natural log-transformed (ln(x + 1)). Associations with complicated malaria were assessed by univariable and multivariable logistic regression (age, parasitaemia, log-CRP, log-PCT), primarily via a complete-case approach, with a collinearity-reduced sensitivity model and multiple imputation by chained equations (20 datasets, pooled by Rubin rules) as pre-specified sensitivity analyses; all estimates are reported as odds ratios with 95% confidence intervals. Because two patients each contributed two episodes, key analyses were repeated after excluding the second episodes (n = 91). Biomarker discrimination was summarised by ROC analysis with 1000-iteration bootstrap confidence intervals and Youden-optimal thresholds (J = sensitivity + specificity − 1). The nine IMSC-NIT items were fixed a priori as the clinical components of the operational composite plus their renal laboratory correlates, together with admission markers discriminating at Youden-optimal thresholds; item selection was deliberately not based on multivariable statistical significance, which is unreliable at this sample size [14,15]. Items were equally weighted (one point each): with 24 to 31 events, data-driven weights would be estimated with substantial uncertainty and would risk overfitting [14,15]; as a sensitivity analysis, an integer-weighted variant assigning two points to the items with the highest univariable discrimination (age and urea) was compared with the equal-weight formulation. Because five items overlap the outcome definition, the full index is subject to incorporation bias [16] and its metrics quantify severity capture rather than independent prognostication; a pre-specified analysis restricted to the four non-overlapping items (age, CRP, PCT, platelet count) therefore provides the primary estimate of discrimination. Missing index items were scored as not met (0), with complete-case and multiply imputed sensitivity analyses. Internal validation used 1000-iteration bootstrap optimism correction with Youden thresholds re-derived within each resample. Calibration (bootstrap calibration slope and intercept, and observed versus expected complication counts by score level [17]) was assessed for the restricted score only, because calibrating the full index against an outcome it partly defines is not meaningful. Two-sided *p* < 0.05 denoted significance.

## 3. Results

### 3.1. Travel Destinations and Patterns

The 93 episodes involved travel to 37 countries across three continents (Figure 1, Table 1). West, East and Central Africa together accounted for 77 of 93 episodes (82.8%; 32.3%, 25.8% and 24.7%, respectively); the remainder had travelled to Southern Africa, Asia (7.5%), the Americas or North Africa. The most frequent countries were Cameroon and Nigeria (12 each) and Tanzania (9), and 12 episodes (12.9%) involved multi-country itineraries. A documented admission year was available for only 50 of 93 episodes, precluding a reliable temporal-trend analysis.

### 3.2. Cohort Characteristics

*Plasmodium falciparum monoinfection was dominant (72/93, 77.4%), with mixed falciparum-containing infections in a further 8 episodes (8.6%; any falciparum 80/93, 86.0%), P. vivax in 11 (11.8%) and unspeciated Plasmodium in 2 (2.2%).* Among the 85 episodes with documented status, 88.2% had used no chemoprophylaxis. At least one chronic comorbidity was documented in 27 of the 75 episodes with available information (36.0%); this field was free text and not systematically structured. The reported day of symptoms at presentation was documented in 36 of 93 episodes (median 5 days, IQR 3–6.25). Fever was near-universal, and the commonest symptoms were chills, headache and myalgia. Admission laboratory values and clinical signs, overall and by group, are shown in Table 2; the four outcome-defining complications are reported separately in Section 3.3 to avoid duplicating the dependent variable.

### 3.3. Complicated Malaria: Frequency and Pattern of Organ Involvement

Complicated malaria occurred in 31 of 93 episodes (33.3%): 13 had a single complication, 13 had two, three had three and two had four concurrently. The most frequent complication was renal failure (24/85 evaluable, 28.2%), followed by macroscopic haemoglobinuria (16/85, 18.8%), jaundice (10/86, 11.6%) and impaired consciousness (6/89, 6.7%). As documented in the clinical records, one patient died (cerebral malaria complicated by septic shock) and three others received organ support (mechanical ventilation or renal replacement therapy) or intensive-care admission during the illness; because these outcomes were captured from free-text notes rather than a dedicated follow-up field, they represent a documented minimum rather than a systematic ascertainment. Applying the WHO 2023 severe-malaria criteria assessable in this dataset (impaired consciousness [Glasgow Coma Scale < 11], severe anaemia [haemoglobin < 7 g/dL], renal impairment [creatinine > 3.0 mg/dL or urea > 20 mmol/L] and hyperparasitaemia [>10%]), 10 of 93 episodes (10.8%) met at least one criterion; the WHO shock criterion could not be applied because no numeric blood-pressure measurements were recorded (only a binary clinician-documented hypotension flag was available), and acidosis, hypoglycaemia, bilirubin-based jaundice and the pulmonary and bleeding criteria were likewise unavailable, so this is a lower bound; the availability and positivity of each WHO criterion are detailed in Table S1. Of the 31 episodes with a complicated course by our operational composite, 8 also met a WHO criterion and 23 did not, whereas 2 WHO-positive episodes fell outside the composite, confirming that the composite is broader than the strictly assessable WHO definition.

### 3.4. Complicated Versus Uncomplicated Malaria: Demographic and Clinical Comparisons

Complicated and uncomplicated cases are compared in Table 2. After Benjamini–Hochberg adjustment, older age, higher urea, higher CRP and hepatomegaly remained significantly associated with complicated disease; procalcitonin, a lower platelet count and creatinine were associated in unadjusted analyses but did not survive correction. Peripheral parasitaemia was not associated with a complicated course.

### 3.5. Discriminative Performance of Admission Biomarkers

In the ROC analysis (Table 3), age and urea showed the highest discrimination for complicated malaria, exceeding the inflammatory markers, platelet count, and creatinine, whereas peripheral parasitaemia was essentially non-discriminative.

### 3.6. Admission Correlates of Complicated Malaria: Logistic Regression

Univariable and multivariable results are shown in Table 4. In univariable regression, older age, log-CRP, log-procalcitonin, urea and creatinine were each associated with complicated malaria, whereas parasitaemia was not; the binary outcome components are not estimable in regression because, by construction, they do not occur in uncomplicated episodes (quasi-complete separation). In the pre-specified multivariable model (complete cases, n = 61, 24 events) age was the only independently associated variable (odds ratio 1.05 per year, 95% CI 1.00–1.10; *p* = 0.049); the inflammatory markers lost significance after mutual adjustment, consistent with their strong collinearity (CRP–procalcitonin Spearman 0.60; variance inflation factors 14.2 and 3.9). A collinearity-reduced sensitivity model (age odds ratio 1.06, 95% CI 1.02–1.11; *p* = 0.009) and multiple imputation by chained equations (n = 93; age odds ratio 1.07, 95% CI 1.02–1.11; *p* = 0.003) both confirmed age as the only independently associated variable, as did the exclusion of the two second episodes (n = 91; age odds ratio 1.05, 95% CI 1.00–1.11; *p* = 0.041).

### 3.7. Derivation of the IMSC-NIT Graded Severity Criteria (Exploratory)

Because the WHO 2023 thresholds were developed for semi-immune endemic populations and were rarely met in this non-immune cohort, we combined, a priori, the clinical components of the operational composite and their renal laboratory correlates (urea, creatinine) with the discriminating admission markers (age, CRP, procalcitonin, platelet count) at Youden-optimal cut-offs into a nine-item graded severity instrument, the Imported Malaria Severity Criteria for Non-Immune Travellers (IMSC-NIT), each item scoring one point (Table 5, Panel A). The thresholds of the six continuous items are the Youden-optimal cut-offs derived from the ROC analyses in Table 3, that is, the value of each marker maximising J = sensitivity + specificity − 1 in this cohort (for example, 43 years is the Youden point of the age ROC curve, with a sensitivity of 67.7% and a specificity of 73.8%); the three clinical items score the presence of the sign. Five of the nine items overlap with, or are correlates of, the composite outcome, so the full index is a standardised severity-capture instrument, not a prognostic model: its apparent area under the curve of 0.88 (95% bootstrap CI 0.80–0.94; optimism-corrected 0.85) quantifies how completely it recapitulates the severity phenotype, and the complication rate rose monotonically with the score (Panel B). Assigning two points to the two items with the highest univariable discrimination (age and urea) did not improve discrimination (AUC 0.875 versus 0.877), supporting the simpler equal-weight formulation. A fully scored index was available in 52 of 93 episodes (55.9%) (per-variable availability in Table S2); items with missing values were scored as not met (0), and the apparent AUC was 0.81 in complete cases (n = 52) and 0.87 (range 0.86–0.88) across 20 multiply imputed datasets. The pre-specified analysis restricted to the four items that do not overlap the outcome provides the primary estimate of discrimination: AUC 0.76 (95% bootstrap CI 0.65–0.85; optimism-corrected 0.72, with thresholds re-derived within each bootstrap resample), compared with 0.72 for age alone, and the complication rate rose monotonically across the restricted score: 4/35 (11.4%) at 0 points, 10/28 (35.7%) at 1, 6/15 (40.0%) at 2, 5/9 (55.6%) at 3 and 6/6 (100%) at 4 points. The restricted score showed acceptable internal calibration (bootstrap calibration slope 1.02, 95% CI 0.64–1.67; calibration intercept 0.04; odds ratio per point 2.41, 95% CI 1.57–3.71; observed versus expected counts by score level in Table S3). Panel C reports the apparent operating characteristics of the full index at derivation-cohort cutpoints; these are derivation-cohort values, require external validation and imply no clinical role. Because strict WHO criteria were met by only 10 of 93 episodes, the graded index is intended to complement the WHO definition by standardising the description of the larger group of non-immune travellers with organ involvement, not to replace it.

Thresholds for the continuous items are the Youden-optimal cut-offs (J = sensitivity + specificity − 1) from the ROC analyses in Table 3; each item scores one point (an integer-weighted variant did not improve discrimination, Section 3.7). Positive and negative predictive values in Panel C are specific to the derivation-cohort prevalence of the broad operational composite (33.3%) and are not transportable to other settings; scoring missing items as not met may additionally inflate the negative predictive value. The apparent area under the curve is 0.88 (bootstrap CI 0.80–0.94; optimism-corrected 0.85), a derivation-cohort value requiring external validation. GCS, Glasgow Coma Scale; PPV/NPV, positive/negative predictive value.

## 4. Discussion

### 4.1. Principal Findings

In this two-centre cohort of 93 imported malaria episodes in 91 adults in Poland, one-third had a complicated course, most often renal failure, followed by macroscopic haemoglobinuria, jaundice and impaired consciousness. The near-universal absence of chemoprophylaxis (88.2% of those with documented status) is a striking and modifiable prevention gap, although the study was not designed or powered to test its association with disease severity. Older age was the only variable independently associated with complicated disease, whereas peripheral parasitaemia showed no association, and complication rates did not differ significantly across sub-Saharan African sub-regions, a finding to be interpreted cautiously given the limited power of this comparison [18]. One patient died and three required organ support or intensive care, underlining that a minority progressed to life-threatening disease. Strict WHO severe-malaria criteria were met in fewer episodes (10 of 93 on the assessable criteria) than our broader composite identified (31 of 93). Because six of the ten WHO criteria could not be assessed in this dataset, this comparison reflects only the assessable criteria (Table S1); within that limit, it is consistent with the possibility that thresholds calibrated for semi-immune endemic populations under-identify the severe end of the spectrum in non-immune travellers. Unlike Western European referral centres, whose imported malaria burden is shaped by post-colonial migration and visiting friends and relatives travel, Poland and other Central and Eastern European countries have no comparable colonial legacy; their cases arise predominantly in non-immune tourists and occupational or missionary travellers, a context that may underlie both the near-universal absence of chemoprophylaxis and the non-immune clinical presentation.

### 4.2. Correlates of Complicated Disease

Age was the dominant predictor across all analyses, consistent with large European cohorts [5,19]. Plausible contributors, none measured here, include immunosenescence and age-associated cytokine dysregulation [20], reduced splenic clearance of parasitised erythrocytes [21], and greater endothelial vulnerability to microvascular sequestration [22]; chronological age probably also proxies for unrecorded comorbidity. CRP, procalcitonin, and urea were elevated in complicated disease but did not remain independent of age, in keeping with the European biomarker literature [10,11,23]. Urea outperformed creatinine (area under the curve 0.75 vs. 0.63), plausibly because urea rises early with pre-renal azotaemia whereas creatinine rises only once established tubular injury develops [24,25], making urea an earlier signal of impending organ compromise. Peripheral parasitaemia was non-discriminative (0.56). Sequestration of mature *P. falciparum* in the deep microvasculature is a biologically plausible explanation [26], although our data cannot demonstrate it directly, and contributions from sample size, missing values, and the timing of sampling cannot be excluded; in practical terms, a low peripheral smear should not be used to exclude complicated disease.

### 4.3. The IMSC-NIT as an Exploratory Severity-Capture Index

Strict WHO 2023 severe-malaria criteria were met by only a minority of this non-immune cohort (10 of 93, 10.8%, on the assessable criteria; median complicated-group creatinine 1.1 mg/dL), fewer than the 31 identified by our broader operational composite, which motivated the recalibrated admission thresholds of the IMSC-NIT. The index is built from routine venous parameters and bedside signs obtainable within the first hour of emergency department assessment, positioned upstream of intensive-care instruments such as the Sequential Organ Failure Assessment score, which require blood-gas and vasopressor data [27,28]; any triage role would first have to be established by prospective external validation. In the restricted analysis, the biomarker items added only modest discrimination beyond age alone (AUC 0.76 versus 0.72), and this incremental value likewise requires confirmation in larger cohorts. Malaria-specific scores such as the Coma Acidosis Malaria score and the Malaria Score for Adults were developed in semi-immune endemic populations and use late-stage thresholds (for example, Glasgow Coma Scale < 11 or creatinine > 3 mg/dL) that are insensitive to the early presentation of non-immune travellers [29,30]. We stress that the IMSC-NIT is a derivation-phase, hypothesis-generating severity-capture index: because five of its nine items overlap the outcome, its apparent discrimination (area under the curve 0.88, optimism-corrected 0.85) reflects how completely it recapitulates the severity phenotype rather than independent prognostication, and external validation is required before clinical adoption.

### 4.4. Strengths and Limitations

This study has important limitations. It is retrospective with incomplete data capture (effective multivariable n = 61; events-per-variable ratio approximately 6), uses clinical rather than strict biochemical outcome definitions, and is limited in power (n = 93 episodes) and generalisability (two referral centres in a single country, with possible referral bias towards more complex cases). Hard clinical outcomes were not systematically ascertained: there were no structured fields for mortality, intensive-care admission, mechanical ventilation, vasopressor use, or renal replacement therapy, such events being identifiable only from free-text notes (a documented minimum), and length of stay was reconstructable in only 43 of 93 episodes; the prognostic significance of the operational composite against hard endpoints therefore could not be tested. Serum bilirubin and baseline or serial creatinine were unavailable, so KDIGO staging and bilirubin-based jaundice definitions could not be applied, and renal impairment and jaundice reflect binary clinical judgements by the treating physicians. Arterial blood gas data were absent, so metabolic acidosis and a respiratory item, relevant to malaria-associated acute respiratory distress syndrome [31], could not be assessed. Comorbidities were recorded as unstructured free text (at least one chronic condition documented in 27 of 75 evaluable episodes), precluding formal adjustment, and age may partly proxy unmeasured comorbidity burden. The reported day of symptoms was documented in only 36 of 93 episodes and treatment initiation dates were unavailable, so diagnostic and treatment delay, a plausible determinant of severity, could not be analysed. Travel destinations rather than confirmed places of acquisition were recorded, and regional assignment for multi-country itineraries is approximate. The IMSC-NIT was derived in a single cohort without external validation; five of its nine items overlap the outcome (incorporation bias [16]), its thresholds are data-derived (residual optimism may persist despite bootstrap correction with per-resample threshold re-derivation), and calibration was assessed only internally and only for the restricted score. Because missing index items were scored as not met, incompletely documented patients received systematically lower scores, which may inflate the apparent negative predictive value; complete-case and multiply imputed analyses gave consistent discrimination. Two patients contributed two episodes each; excluding the second episodes did not materially change the results. Macroscopic haemoglobinuria was defined broadly, although all cases were documented before treatment, excluding post-artesunate delayed haemolysis [32].

### 4.5. Future Directions

The IMSC-NIT requires prospective, multi-centre external validation with formal calibration and pre-specified hard clinical endpoints (mortality, intensive-care admission, organ support), ideally through networks such as TropNetEurop, together with an adequately powered head-to-head comparison with the WHO criteria and existing malaria severity scores. Future data collection should capture serum bilirubin, arterial blood gases, numeric blood pressure, venous lactate, structured comorbidities and the timing of diagnosis and treatment. Targeted pre-travel counselling to close the chemoprophylaxis gap is a further priority.

## 5. Conclusions

In this 11-year two-centre cohort of 93 imported malaria episodes in 91 adults in Poland, one-third had a complicated course, acquired overwhelmingly in sub-Saharan Africa, and nearly nine in ten had used no chemoprophylaxis. Older age was the only variable independently associated with a complicated course, and parasitaemia was non-discriminative. Because the WHO 2023 severe-malaria criteria performed poorly in this non-immune population, we derived the exploratory IMSC-NIT severity-capture criteria. Because five of the nine items overlap the outcome, the full index functions as a severity-capture instrument (apparent area under the curve 0.88, optimism-corrected 0.85) rather than a prognostic model; in the pre-specified analysis restricted to the four non-overlapping items, discrimination was moderate (0.76; optimism-corrected 0.72). External validation is required before any clinical use.

## Figures and Tables

**Table 1:** Travel destinations ranked by number of episodes (n = 93).

Country	n	Country	n
Cameroon	12	Pakistan	2
Nigeria	12	Sierra Leone	2
Tanzania	9	Sudan	2
Kenya	6	Zambia	2
Uganda	6	Angola	1
Benin	4	Brazil	1
Congo	4	Burkina Faso	1
DRC	4	Colombia	1
Ghana	4	Dominican Rep.	1
Guinea	4	Ethiopia	1
Senegal	4	Madagascar	1
Togo	4	Malawi	1
CAR	3	Mauritania	1
Côte d’Ivoire	3	Mauritius	1
Gambia	3	Morocco	1
India	3	Mozambique	1
Liberia	3	Thailand	1
South Africa	3	Western Sahara	1
Guinea-Bissau	2		

DRC = Democratic Republic of the Congo; CAR = Central African Republic. Counts sum to 115 rather than 93 because patients with a multi-country itinerary are counted once for each country visited; one further patient had only “South-East Asia” recorded and could not be assigned to a country.

**Table 2:** Cohort characteristics overall and stratified by complicated vs. uncomplicated malaria.

Variable	Overall (n = 93)	Uncomplicated (n = 62)	Complicated (n = 31)	*p* (FDR-adj)
**Demographics**				
Age (years), median (IQR)	40 (30–48)	36 (28–44)	47 (38–53)	0.0008 (0.005)
Male sex, n (%)	72 (77.4)	47 (75.8)	25 (80.6)	0.79
**Aetiology**				
*P. falciparum* monoinfection, n (%)	72 (77.4)	46 (74.2)	26 (83.9)	0.43
* P. vivax*, n (%)	11 (11.8)	9 (14.5)	2 (6.5)	0.33
Mixed (*falciparum*-containing), n (%)	8 (8.6)	5 (8.1)	3 (9.7)	1.00
Unspeciated *Plasmodium*, n (%)	2 (2.2)	2 (3.2)	0 (0.0)	0.55
No prophylaxis, n/N (%)	75/85 (88.2)	49/57 (86.0)	26/28 (92.9)	0.49
**Laboratory markers, median (IQR)**				
Parasitaemia (%)	1.0 (0.2–4.0)	1.0 (0.2–3.8)	1.8 (0.2–4.6)	0.38
Haemoglobin (g/dL)	12.8 (11.1–14.0)	12.8 (11.7–13.9)	12.9 (10.2–14.2)	0.88
Platelet count (×10^3^/µL)	68 (45–115)	75 (52–115)	50 (34–97)	0.031 (0.088)
C-reactive protein (mg/L)	113 (55–163)	94 (42–150)	147 (86–190)	0.007 (0.036)
Procalcitonin (ng/mL)	2.1 (0.6–5.4)	1.8 (0.2–4.8)	2.6 (1.5–20.7)	0.019 (0.065)
Urea (mg/dL)	28 (22–41)	27 (22–33)	40 (26–64)	0.0005 (0.005)
Creatinine (mg/dL)	1.0 (0.8–1.1)	1.0 (0.8–1.1)	1.1 (0.8–1.4)	0.041 (0.103)
ALT (U/L)	49 (31–92)	63 (32–99)	38 (27–77)	0.17
AST (U/L)	55 (31–99)	50 (32–116)	59 (27–89)	0.67
**Symptoms and signs, n/N (%)**				
Chills	47/81 (58.0)	33/55 (60.0)	14/26 (53.8)	0.64
Headache	43/79 (54.4)	28/55 (50.9)	15/24 (62.5)	0.46
Myalgia	31/79 (39.2)	23/56 (41.1)	8/23 (34.8)	0.80
Hepatomegaly	19/83 (22.9)	8/57 (14.0)	11/26 (42.3)	0.010 (0.038)
Splenomegaly	34/83 (41.0)	20/57 (35.1)	14/26 (53.8)	0.15

Values are median (IQR) or n (%). *p*-values from Mann–Whitney U test (continuous) or Fisher’s exact test (categorical); FDR-adjusted *p*-values (Benjamini–Hochberg across 20 biomarker/sign comparisons) in parentheses where applied. Aetiology *p*-values are from the Fisher exact test, each category versus all other categories (overall 4 × 2 chi-square *p* = 0.49).

**Table 3:** Item-level discriminative characteristics of candidate admission markers and IMSC-NIT items.

Biomarker	AUC (95% CI)	Optimal Threshold	Sensitivity	Specificity	n	Incorporation Status
Age (years)	0.72 (0.60–0.81)	≥43	67.7%	73.8%	92	Independent item
Urea (mg/dL)	0.75 (0.62–0.88)	>33	73.1%	73.9%	72	Correlate of renal outcome component
C-reactive protein (mg/L)	0.68 (0.56–0.79)	>157	46.7%	81.4%	89	Independent item
Procalcitonin (ng/mL)	0.67 (0.53–0.81)	>8.9	42.3%	93.6%	73	Independent item
Platelet count (×10^3^/µL)	0.64 (0.50–0.76)	<57	58.1%	71.0%	93	Independent item
Creatinine (mg/dL)	0.63 (0.50–0.76)	>1.1	51.7%	79.0%	91	Correlate of renal outcome component
Parasitaemia (%)	0.56 (0.42–0.69)	>7.5	25.0%	89.8%	77	Tested, not retained
Impaired consciousness (GCS < 15)	Not defined *	GCS < 15	21.4%	100% ‡	89	Outcome component
Macroscopic haemoglobinuria	Not defined *	Present	57.1%	100% ‡	85	Outcome component
Clinical jaundice	Not defined *	Present	35.7%	100% ‡	86	Outcome component

AUC = area under the ROC curve; 95% CI by 1000-iteration bootstrap. Optimal thresholds by Youden index (J = sensitivity + specificity − 1). * A classical ROC curve is not defined for a dichotomous item, which yields a single operating point; sensitivity is reported on evaluable episodes. ‡ Specificity is 100% by construction because these items are components of the outcome definition; they are shown for transparency and excluded from the restricted four-item analysis.

**Table 4:** Univariable and multivariable logistic regression for complicated malaria.

Variable	Univariable OR (95% CI)	*p*	Multivariable OR (95% CI) †	*p*	MICE OR (95% CI) ‡	*p*
Age (per year)	1.07 (1.02–1.11)	0.002	1.05 (1.00–1.10)	0.049	1.07 (1.02–1.11)	0.003
Parasitaemia (per %)	1.06 (0.94–1.19)	0.33	1.06 (0.89–1.27)	0.49	1.05 (0.91–1.22)	0.47
CRP (per mg/L)	1.009 (1.00–1.02)	0.007	1.72 (0.67–4.45) §	0.26	1.53 (0.80–2.89) §	0.20
Procalcitonin (per ng/mL)	1.09 (1.02–1.16)	0.013	1.37 (0.73–2.58) §	0.32	1.35 (0.81–2.23) §	0.25
Urea (per mg/dL)	1.07 (1.02–1.11)	0.004	–	–	–	–
Creatinine (per mg/dL)	4.12 (1.11–15.26)	0.034	–	–	–	–
Platelets (per 10^3^/µL)	0.996 (0.99–1.00)	0.21	–	–	–	–
Haemoglobin (per g/dL)	0.93 (0.77–1.14)	0.50	–	–	–	–
Impaired consciousness (GCS < 15)	Not estimable (outcome component)	–	–	–	–	–
Macroscopic haemoglobinuria	Not estimable (outcome component)	–	–	–	–	–
Clinical jaundice	Not estimable (outcome component)	–	–	–	–	–
Renal impairment (clinician flag)	Not estimable (outcome component)	–	–	–	–	–

† Complete-case model (age, parasitaemia, log-CRP, log-PCT; n = 61, 24 events). ‡ Multiple imputation by chained equations (n = 93; 20 datasets pooled by Rubin rules). § CRP and procalcitonin entered multivariable models as ln(x + 1); odds ratios are per unit of the log-transformed marker. Collinearity-reduced sensitivity model (age, parasitaemia, log-CRP; n = 74, 27 events): age OR 1.06 (95% CI 1.02–1.11; *p* = 0.009). Binary outcome components are not estimable owing to quasi-complete separation (by construction absent in uncomplicated episodes).

**Table 5:** IMSC-NIT (Imported Malaria Severity Criteria for Non-Immune Travellers): scoring rubric and derivation-cohort characteristics.

**Panel A. Scoring Criteria: One Point per Criterion Met (Total 0–9).**					
**#**	**Variable**	**Positive if**		**Pts**	
1	Age	≥43 years		1	
2	Blood urea	>33 mg/dL		1	
3	Serum creatinine	>1.1 mg/dL		1	
4	C-reactive protein (CRP)	>157 mg/L		1	
5	Procalcitonin (PCT)	>8.9 ng/mL		1	
6	Platelet count	<57 × 10^3^/µL		1	
7	Consciousness (GCS)	<15		1	
8	Macroscopic haemoglobinuria	Present on admission		1	
9	Clinical jaundice	Present on admission		1	
	**TOTAL**	**Sum of points (range 0–9)**		**0–9**	
**Panel B. Score Distribution and Complication Rate (n = 93)**					
**IMSC-NIT score**		**Episodes, n**		**Complicated, n (%)**	
**0–1**		45		3 (6.7)	
**2–3**		28		11 (39.3)	
**≥4**		20		17 (85.0)	
**Panel C. Diagnostic Performance at Score Cutpoints (Apparent On Derivation Cohort)**					
**Cutpoint**	**Sensitivity**	**Specificity**	**PPV**		**NPV**
**≥2**	90.3%	67.7%	58.3%		93.3%
**≥3**	71.0%	83.9%	68.8%		85.2%
**≥4**	54.8%	95.2%	85.0%		80.8%

## Data Availability

The data presented in this study are available on request from the corresponding author. The data are not publicly available owing to privacy and ethical restrictions concerning patient information. The analysis code is available from the corresponding author on request.

## Supplementary Materials

The following supporting information can be downloaded at: https://www.mdpi.com/article/10.3390/jcm15187269/s1, Figure S1: Selection of episodes and analysis subsets; Table S1: Availability and positivity of the WHO 2023 severe-malaria criteria in the dataset (n = 93 episodes); Table S2: Per-variable data availability (n = 93 episodes); Table S3: Sensitivity analyses: missing-data handling and statistical independence; STROBE and TRIPOD checklists.

## References

[1] World Health Organization (2024). World Malaria Report 2024.

[2] European Centre for Disease Prevention and Control (2024). Malaria: Annual Epidemiological Report for 2022.

[3] Stępień M. (2019). Malaria in Poland in 2014–2018. Przegl. Epidemiol..

[4] Mischlinger J., Rönnberg C., Álvarez-Martínez M.J., Bühler S., Paul M., Schlagenhauf P., Petersen E., Ramharter M. (2020). Imported Malaria in Countries Where Malaria Is Not Endemic: A Comparison of Semi-Immune and Nonimmune Travelers. Clin. Microbiol. Rev..

[5] Bruneel F., Tubach F., Mira J.-P., Houze S., Gibot S., Huisse M.-G., Megarbane B., Choquet C., Corne P., Peytel E. (2016). Imported Falciparum Malaria in Adults: Host- and Parasite-Related Factors Associated with Severity. The French Prospective Multicenter PALUREA Cohort Study. Intensive Care Med..

[6] Seringe E., Thellier M., Fontanet A., Legros F., Bouchaud O., Ancelle T., Kendjo E., Houze S., Le Bras J., Danis M. (2011). Severe Imported *Plasmodium falciparum* Malaria, France, 1996–2003. Emerg. Infect. Dis..

[7] World Health Organization (2023). WHO Guidelines for Malaria, 14 March 2023.

[8] Tatem A.J., Jia P., Ordanovich D., Falkner M., Huang Z., Howes R., Hay S.I., Gething P.W., Smith D.L. (2017). The Geography of Imported Malaria to Non-Endemic Countries: A Meta-Analysis of Nationally Reported Statistics. Lancet Infect. Dis..

[9] Schlagenhauf P., Weld L., Goorhuis A., Gautret P., Weber R., von Sonnenburg F., Lopez-Vélez R., Jensenius M., Cramer J.P., Field V.K. (2015). Travel-Associated Infection Presenting in Europe (2008–12): An Analysis of EuroTravNet Longitudinal, Surveillance Data, and Evaluation of the Effect of the Pre-Travel Consultation. Lancet Infect. Dis..

[10] Hesselink D.A., Burgerhart J.S., Bosmans-Timmerarends H., Petit P., van Genderen P.J.J. (2009). Procalcitonin as a Biomarker for Severe *Plasmodium falciparum* Disease: A Critical Appraisal of a Semi-Quantitative Point-of-Care Test in a Cohort of Travellers with Imported Malaria. Malar. J..

[11] Balerdi-Sarasola L., Parolo C., Fleitas P., Cruz A., Subirà C., Rodríguez-Valero N., Almuedo-Riera A., Letona L., Álvarez-Martínez M.J., Valls M.E. (2023). Host Biomarkers for Early Identification of Severe Imported *Plasmodium falciparum* Malaria. Travel Med. Infect. Dis..

[12] Collins G.S., Reitsma J.B., Altman D.G., Moons K.G.M. (2015). Transparent Reporting of a Multivariable Prediction Model for Individual Prognosis or Diagnosis (TRIPOD): The TRIPOD Statement. BMJ.

[13] von Elm E., Altman D.G., Egger M., Pocock S.J., Gøtzsche P.C., Vandenbroucke J.P. (2007). The Strengthening the Reporting of Observational Studies in Epidemiology (STROBE) Statement: Guidelines for Reporting Observational Studies. Lancet.

[14] Heinze G., Wallisch C., Dunkler D. (2018). Variable Selection: A Review and Recommendations for the Practicing Statistician. Biom. J..

[15] Riley R.D., Ensor J., Snell K.I.E., Harrell F.E., Martin G.P., Reitsma J.B., Moons K.G.M., Collins G., van Smeden M. (2020). Calculating the Sample Size Required for Developing a Clinical Prediction Model. BMJ.

[16] Rutjes A.W.S., Reitsma J.B., Di Nisio M., Smidt N., van Rijn J.C., Bossuyt P.M.M. (2006). Evidence of Bias and Variation in Diagnostic Accuracy Studies. Can. Med. Assoc. J..

[17] Van Calster B., McLernon D.J., van Smeden M., Wynants L., Steyerberg E.W., on behalf of Topic Group ‘Evaluating diagnostic tests and prediction models’ of the STRATOS initiative (2019). Calibration: The Achilles Heel of Predictive Analytics. BMC Med..

[18] Duffy F., Bernabeu M., Babar P.H., Kessler A., Wang C.W., Vaz M., Chery L., Mandala W.L., Rogerson S.J., Taylor T.E. (2019). Meta-Analysis of *Plasmodium falciparum* var Signatures Contributing to Severe Malaria in African Children and Indian Adults. mBio.

[19] Legros F., Bouchaud O., Ancelle T., Arnaud A., Cojean S., Le Bras J., Danis M., Fontanet A., Durand R., Autochthonous Malaria Epidemiology (2007). Risk Factors for Imported Fatal *Plasmodium falciparum* Malaria, France, 1996–2003. Emerg. Infect. Dis..

[20] Baylis D., Bartlett D.B., Patel H.P., Roberts H.C. (2013). Understanding How We Age: Insights into Inflammaging. Longev. Heal..

[21] Buffet P.A., Safeukui I., Deplaine G., Brousse V., Prendki V., Thellier M., Turner G.D., Mercereau-Puijalon O. (2011). The Pathogenesis of *Plasmodium falciparum* Malaria in Humans: Insights from Splenic Physiology. Blood.

[22] Ungvari Z., Tarantini S., Donato A.J., Galvan V., Csiszar A. (2018). Mechanisms of Vascular Aging. Circ. Res..

[23] D’Abramo A., Rinaldi F., Vita S., Mazzieri R., Corpolongo A., Palazzolo C., Ascoli Bartoli T., Faraglia F., Giancola M.L., Girardi E. (2024). A Machine Learning Approach for Early Identification of Patients with Severe Imported Malaria. Malar. J..

[24] Lameire N., Van Biesen W., Vanholder R. (2005). Acute Renal Failure. Lancet.

[25] Carvounis C.P., Nisar S., Guro-Razuman S. (2002). Significance of the Fractional Excretion of Urea in the Differential Diagnosis of Acute Renal Failure. Kidney Int..

[26] Dondorp A.M., Desakorn V., Pongtavornpinyo W., Sahassananda D., Silamut K., Chotivanich K., Newton P.N., Pitisuttithum P., Smithyman A.M., White N.J. (2005). Estimation of the Total Parasite Biomass in Acute Falciparum Malaria from Plasma PfHRP2. PLoS Med..

[27] Bruneel F., Tubach F., Corne P., Megarbane B., Mira J.-P., Peytel E., Camus C., Schortgen F., Azoulay E., Cohen Y. (2010). Severe Imported Falciparum Malaria: A Cohort Study in 400 Critically Ill Adults. PLoS ONE.

[28] Teparrukkul P., Hantrakun V., Imwong M., Teerawattanasook N., Wongsuvan G., Day N.P.J., Dondorp A.M., West T.E., Limmathurotsakul D. (2019). Utility of qSOFA and Modified SOFA in Severe Malaria Presenting as Sepsis. PLoS ONE.

[29] Hanson J., Lee S.J., Mohanty S., Faiz M.A., Anstey N.M., Charunwatthana P., Bin Yunus E., Mishra S.K., Tjitra E., Price R.N. (2010). A Simple Score to Predict the Outcome of Severe Malaria in Adults. Clin. Infect. Dis..

[30] Mishra S.K., Panigrahi P., Mishra R., Mohanty S. (2007). Prediction of Outcome in Adults with Severe Falciparum Malaria: A New Scoring System. Malar. J..

[31] Van den Steen P.E., Deroost K., Deckers J., Van Herck E., Struyf S., Opdenakker G. (2013). Pathogenesis of Malaria-Associated Acute Respiratory Distress Syndrome. Trends Parasitol..

[32] Rolling T., Agbenyega T., Krishna S., Kremsner P.G., Cramer J.P. (2015). Delayed Haemolysis after Artesunate Treatment of Severe Malaria: Review of the Literature and Perspective. Travel Med. Infect. Dis..

