# Supplementary material for: Clinical Spectrum and Admission Correlates of Complicated Imported Malaria in Polish Travellers: An 11-Year Retrospective Analysis with Geographic Mapping of Travel Destinations
Source: J Clin Med. 2026 Sep 18;15(18):7269. doi: 10.3390/jcm15187269 (PMC13607098; doi:10.3390/jcm15187269)
Supplement: Supplementary file 1 [file jcm-15-07269-s001.zip › jcm-4486497-supplementary.pdf]

Supplementary Materials

Clinical spectrum and admission correlates of complicated imported malaria in Polish travellers: an 11-year retrospective analysis with geographic mapping of travel destinations

Contents: Table S1 (availability and positivity of WHO 2023 severe-malaria criteria); Table S2 (per-variable data availability); Table S3 (sensitivity analyses); Figure S1 (episode selection flow diagram). The completed STROBE and TRIPOD checklists are provided as separate files.

Table S1. Availability and positivity of the WHO 2023 severe-malaria criteria in the dataset (n = 93 episodes).

WHO 2023 criterion	Assessable, n/93	Positive, n
**Impaired consciousness (GCS < 11)**	89	4
**Severe anaemia (haemoglobin < 7 g/dL)**	90	2
**Renal impairment (creatinine > 3 mg/dL or urea > 20 mmol/L)**	92	3
**Hyperparasitaemia (> 10%)**	77	4
**Shock (blood-pressure criterion)**	0 (numeric blood pressure not recorded)	Not assessable
**Metabolic acidosis**	0 (no blood-gas data)	Not assessable
**Hypoglycaemia**	0 (admission glucose not systematically recorded)	Not assessable
**Jaundice (bilirubin-based)**	0 (serum bilirubin not recorded)	Not assessable
**Pulmonary oedema / ARDS criterion**	0 (no blood-gas or imaging fields)	Not assessable
**Abnormal bleeding**	0 (no structured field)	Not assessable

At least one assessable criterion was positive in 10 of 93 episodes (10.8%). Of the 31 episodes meeting the operational composite, 8 were WHO-positive and 23 were not; 2 WHO-positive episodes did not meet the composite. Because six of the ten criteria were not assessable, the WHO-positive count is a lower bound and the comparison with the operational composite reflects only the assessable criteria.

Table S2. Per-variable data availability (canonical analysis parsing; n = 93 episodes).

Variable	Available, n/93	Missing, %
**Age**	92	1.1
**Parasitaemia**	77	17.2
**Haemoglobin**	90	3.2
**Platelet count**	93	0.0
**ALT**	91	2.2
**AST**	78	16.1
**C-reactive protein**	89	4.3
**Procalcitonin**	73	21.5
**Urea**	72	22.6
**Creatinine**	91	2.2
**Glasgow Coma Scale**	89	4.3
**Renal impairment (clinician**	85	8.6

**flag)**		
**Clinical jaundice**	86	7.5
**Macroscopic haemoglobinuria**	85	8.6
**Chemoprophylaxis status**	85	8.6
**Reported day of symptoms**	36	61.3
**Sex**	93	0.0
**Chills**	81	12.9
**Headache**	79	15.1
**Myalgia**	79	15.1
**Hepatomegaly**	83	10.8
**Splenomegaly**	83	10.8
**Chronic comorbidity (free text)**	75	19.4

All nine IMSC-NIT items were available in 52 of 93 episodes (55.9%); 24 episodes were missing one item, 11 two items, 3 three items and 3 four items. Missingness was driven mainly by urea (22.6%) and procalcitonin (21.5%).

Table S3. Sensitivity analyses: missing-data handling and statistical independence.

Analysis	n	AUC
**Nine-item IMSC-NIT, missing items scored as not met (primary presentation)**	93	0.88 (95% CI 0.80-0.94; optimism-corrected 0.85)
**Nine-item IMSC-NIT, complete cases only**	52 (17 complicated)	0.81
**Nine-item IMSC-NIT, multiply imputed items (20 datasets)**	93	mean 0.87 (range 0.86-0.88)
**Restricted four-item score, missing items scored as not met (primary discrimination estimate)**	93	0.76 (95% CI 0.65-0.85; optimism-corrected 0.72)
**Restricted four-item score, complete cases only**	71	0.72
**Restricted four-item score, multiply imputed items (20 datasets)**	93	mean 0.74
**Age alone (continuous)**	92	0.72 (95% CI 0.60-0.81)
**Unique patients only (second episodes of two patients excluded): nine-item / restricted**	91	0.88 / 0.76
**Unique patients only: multivariable age odds ratio**	60 complete cases	OR 1.05 per year (95% CI 1.00-1.11), p = 0.041

Restricted four-item score: age >= 43 years, CRP > 157 mg/L, procalcitonin > 8.9 ng/mL, platelets < 57 x 10^3/uL (one point each). Internal calibration of the restricted score: bootstrap calibration slope 1.02 (95% CI 0.64-1.67), intercept 0.04; odds ratio per point 2.41 (95% CI 1.57-3.71). Observed versus expected complicated episodes by restricted-score level (expected counts from the logistic calibration model): 0 points, 4 vs 4.8; 1 point, 10 vs 7.7; 2 points, 6 vs 7.2; 3 points, 5 vs 6.2; 4 points, 6 vs 5.1.

Figure S1. Selection of episodes and analysis subsets.

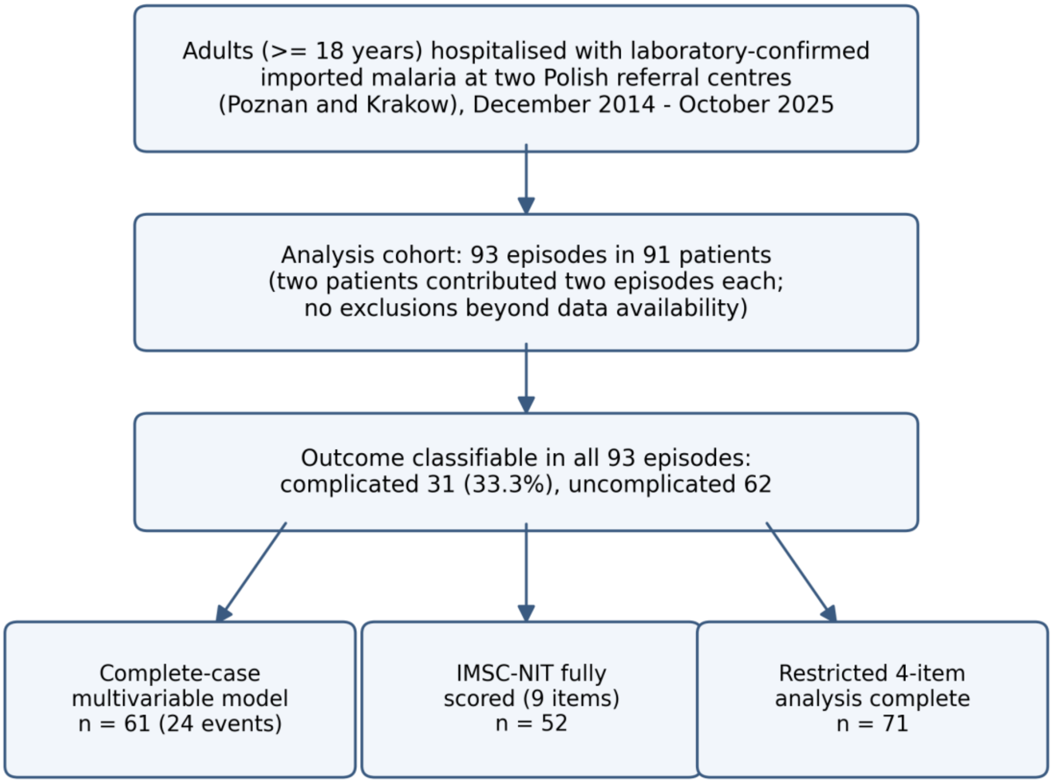

Missing items scored as not met (0) in primary index analyses; complete-case and multiply imputed sensitivity analyses in Table S3.
